# Purinergic enzymes on extracellular vesicles: immune modulation on the go

**DOI:** 10.3389/fimmu.2024.1362996

**Published:** 2024-02-15

**Authors:** Riekje Winzer, Du Hanh Nguyen, Felix Schoppmeier, Filippo Cortesi, Nicola Gagliani, Eva Tolosa

**Affiliations:** ^1^ Department of Immunology, University Medical Center Hamburg-Eppendorf, Hamburg, Germany; ^2^ I. Department of Medicine, University Medical Center Hamburg-Eppendorf, Hamburg, Germany; ^3^ Department of General, Visceral and Thoracic Surgery, University Medical Center Hamburg-Eppendorf, Hamburg, Germany

**Keywords:** extracellular vesicles, purinergic signaling, CD73, adenosine, immune regulation, tumor microenvironment

## Abstract

An increase in the extracellular concentration of ATP as a consequence of cellular stress or cell death results in the activation of immune cells. To prevent inflammation, extracellular ATP is rapidly metabolized to adenosine, which deploys an anti-inflammatory signaling cascade upon binding to P1 receptors on immune cells. The ectonucleotidases necessary for the degradation of ATP and generation of adenosine are present on the cell membrane of many immune cells, and their expression is tightly regulated under conditions of inflammation. The discovery that extracellular vesicles (EVs) carry purinergic enzyme activity has brought forward the concept of EVs as a new player in immune regulation. Adenosine-generating EVs derived from cancer cells suppress the anti-tumor response, while EVs derived from immune or mesenchymal stem cells contribute to the restoration of homeostasis after infection. Here we will review the existing knowledge on EVs containing purinergic enzymes and molecules, and discuss the relevance of these EVs in immune modulation and their potential for therapy.

## 1 Introduction

**Figure 1:**
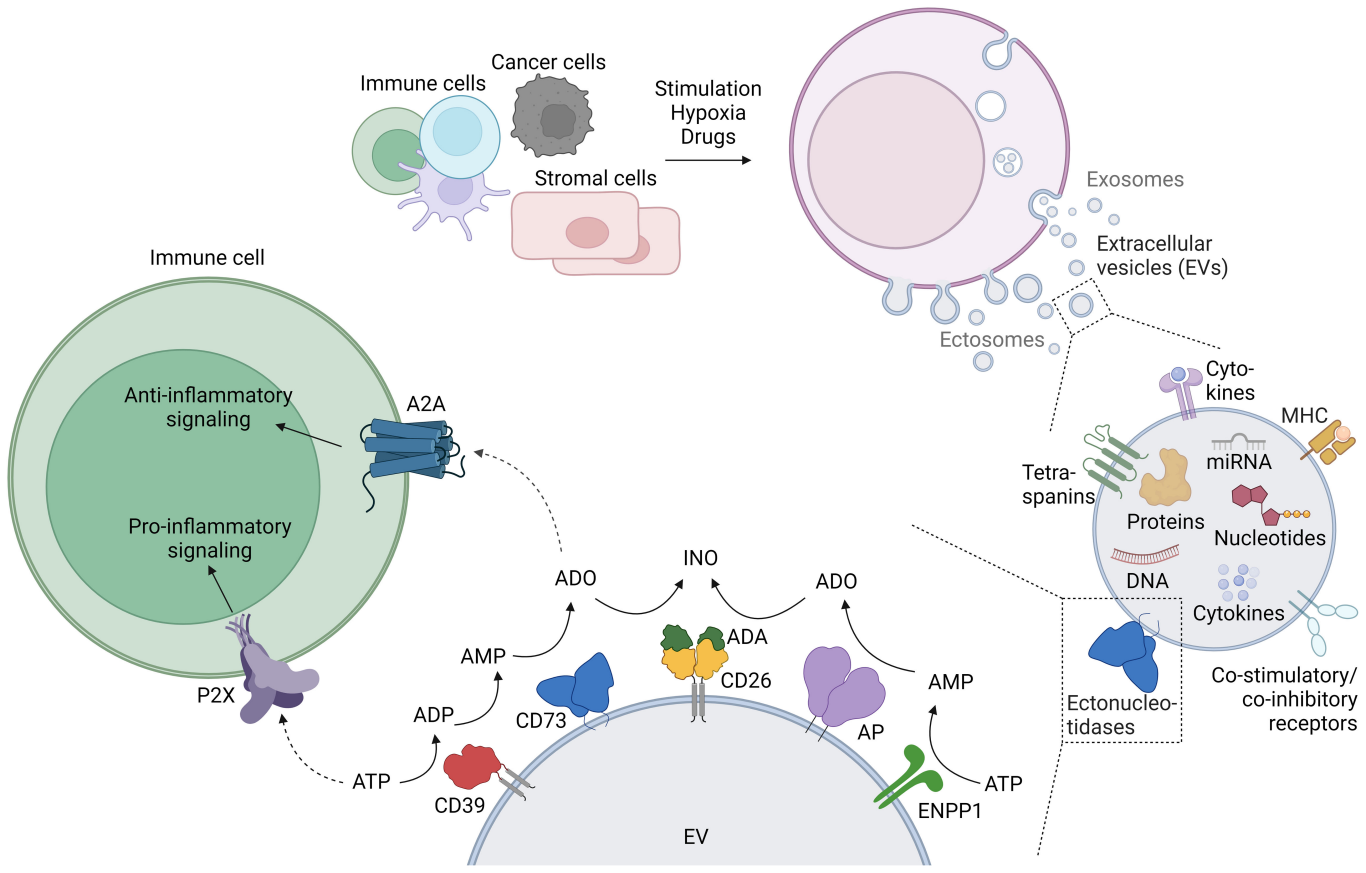
Generation of extracellular vesicles (EVs), their purinergic cargo, and the effect of EV-bound purinergic molecules on immune cells. The release of EVs is physiologic in most cells, and diverse stimuli such as activation or hypoxia enhance EV release. EVs contain a variety of cargo molecules, including purinergic enzymes (ectonucleotidases) at the membrane, and nucleotides enclosed within the lipid bilayer. The enzymatic activity of EV-bound ectonucleotidases can alter the concentration of extracellular adenine nucleotides, which can be sensed by immune cells. This figure was created with BioRender.com.

The adaptive immune system reacts to infected and tumor cells by mounting a tailored response that involves the activation of cytotoxicity mechanisms for the killing of target cells, and the release of inflammatory cytokines that will recruit and activate other immune cells. The aftermath of this response can lead to inflammation, and activated effector cells are tightly controlled by regulatory T cells (Tregs), and by processes such as activation-induced cell death or fratricide. Inflammation, cellular stress and cell death promote the release of adenosine triphosphate (ATP) into the extracellular space, drastically increasing the concentration by several orders of magnitude to the 100 µM range. Extracellular ATP further stimulates immune cells by binding and activating P2 receptors, but can also be metabolized by purinergic enzymes to generate immune suppressive adenosine. The purinergic enzymes involved in the “canonical” degradation of ATP to adenosine are the ectonucleotidases CD39 and CD73. CD39 degrades ATP first to ADP and then further to AMP, and CD73 hydrolyzes AMP to adenosine, which then exerts anti-inflammatory effects by binding to and activating A2A adenosine receptors on immune cells (1). Also the concerted action of other enzymes, such as ENPP1 and alkaline phosphatases (AP) metabolize ATP to adenosine, while adenosine deaminase (ADA) reduces adenosine-mediated signaling by degrading adenosine to inosine (**Figure 1**). The immediate effects upon engagement of purinergic receptors together with the swift activity of the ectoenzymes degrading ATP and generating and degrading adenosine procures a highly efficient system for the regulation of ongoing immune responses.

With a vast range of surface molecules and bioactivities as cargo, extracellular vesicles (EVs) constitute a powerful means of intercellular communication. EVs carry a broad variety of proteins, DNA, RNA and nucleotides on their surface or inside the EV lipid bilayer. This cargo can be transported and exert its function on cells that are located at significant distances from the donor cells. Importantly, inflammation and the tumor environment foster the release of EVs and influence the mechanism of vesicular release and their cargo. Consequently, it is not surprising that EVs can modulate the immune response by playing both pro-inflammatory and anti-inflammatory roles.

Many purinergic molecules are found in EVs. To name a few, the ectonucleotidases CD39, CD73, and ENPP1, APs, and also ADA and its ‘membrane anchor’ CD26. Moreover, both ATP and adenosine were found in EVs. In this review, we will exclusively focus on the immunoregulatory role of EVs containing molecules of the purinergic pathway. For the interested reader, excellent reviews covering the role of EVs in the immune system (2) and the interplay between purinergic signaling and EVs (3) have been recently published.

## 2 Purinergic enzymes and metabolites in extracellular vesicles

EVs are small lipid bilayer particles released by almost all cell types under physiological and pathological conditions. The term EVs comprises different types of vesicles, broadly subdivided into exosomes and ectosomes (**Figure 1**). Exosomes are usually small vesicles (30 – 150 nm) of endosomal origin. They are generated by inward budding of the cell membrane, subsequent formation of multivesicular bodies (MVBs), and release through fusion with the plasma membrane. Ectosome is the generic term for EVs generated by vesicle budding from the plasma membrane. Ectosomes include a variety of vesicles, e.g. microvesicles and apoptotic bodies, and consequently vary greatly in size (100 nm – 2 µm). There are many more types of vesicles, sometimes named after their cell of origin (e.g. oncosomes are ectosomes derived from tumor cells), or their location of release (e.g. trans-synaptic vesicles are released during the formation of the immune synapse), reflecting the huge diversity of EVs (2, 4). Membrane or membrane-associated proteins such as tetraspanins (CD9, CD63, CD81) and Flotillin are typical markers for EVs, although not specific for a certain type of EV. LAMP-1, TSG-101, and Alix are rather related to vesicles derived from the endosomal pathway, and can be used to distinguish exosomes from ectosomes (5). Although EV production by cells occurs under steady state conditions, cell stimulation massively increases EV release: Immune cells release EVs upon activation (6, 7) or light exposure (8). Cancer cells release EVs under hypoxic conditions, photodynamic treatment, chemotherapy, high cytosolic Ca^2+^ concentrations, treatment with cytokines, and stimulation with ATP (9–11). Cytokines, hypoxia and drugs trigger EV release by other cells (e.g. adipocytes, HEK cells) (12). In summary, the release of EVs with different sizes and origins is a physiological occurrence that is enhanced under activation and cellular stress.

The membrane proteins and molecular cargo of the EVs often mirror their cells of origin. In addition to the omnipresent tetraspanins, proteins decorating the surface of the EVs include adhesion molecules, major histocompatibility complex (MHC) proteins (13), immune receptors (14), cytokines (15) and enzymes, among them ectonucleotidases and proteases (2). These proteins often form functional units such as the synaptosome (16) or antigen presentation modules capable of eliciting T cell responses (13). Inside the lipid bilayer, EVs contain cytokines and growth factors (17), cytoskeleton components and enzymes, RNA (including micro RNA and long non-coding RNA) (18, 19), DNA (20), telomeres (7), and nucleotides (21–23) (**Figure 1**).

In 2007, the finding of CD39 and CD73 on the cell membrane of murine Tregs and the generation of immune suppressive adenosine led Deaglio and colleagues to propose a novel mechanism of T cell suppression by Tregs (24). Subsequent reports of tumor- and Treg-derived exosomes expressing CD39 and CD73, which were capable of generating adenosine and immune suppression pioneered research on EVs and purinergic enzymes (25, 26).

The AMPase CD73 is often present in modulatory EVs (**Table 1**). The nature of CD73 as lipid raft-associated, GPI-anchored protein seems to favor a release in vesicles, as EV formation is associated with lipid rafts (53). Therefore, CD73 is even mentioned as EV marker in the “Minimal Information for Studies of Extracellular Vesicles” (MISEV) guidelines (54). Mesenchymal stem cells (MSC) and tumor cells express high levels of CD73, and this is reflected in the corresponding EVs. CD39 is found in EVs derived from acute myeloid leukemia (AML), myeloma, and some solid tumors. Murine Treg-derived EVs harbor both CD39 and CD73, while human Tregs, with very low CD73 on their cell surface, produce EVs with CD39, but no CD73. Because both enzymes are required for the generation of adenosine, it is often the case that one of the enzymatic activities is provided in trans, by neighboring cells. For instance, CD73^+^ EVs rely on the presence of CD39 on the membrane of activated (and sometimes exhausted) T cells to generate adenosine (33), and the suppressive function of human Tregs is lower if responder cells are CD73-negative (31).

**Table 1:** Immunomodulatory function of EVs containing purinergic enzymes, receptors and metabolites.

Purinergic molecule	Cell type	Source	Function	References
CD73	Cancer cells	Human HNSCC cells	Immunosuppressive; tumor-associated macrophages phagocytose HNSCC-derived CD73^+^ EVs promoting tumor immunosuppression, M2 polarization and higher immune checkpoint expression *in vivo*	(27)
		Human and murine glioblastoma cells	Immunosuppressive; T cells take up glioblastoma-derived CD73^+^ EVs, which prevent their expansion, promoting tumor growth *in vivo*, A2A receptor-dependent	(28)
		Ovarian cancer-, colon cancer-, non-small cell lung carcinoma cells	Anti-CD73 antibodies revoke adenosine-mediated immune suppression by tumor-derived EVs *in vitro*	(29)
		Serum of patients with melanoma	Immunosuppressive; CD73^+^ EVs suppress T cell function *in vitro*	(30)
	Immune cells	Activated human peripheral CD8^+^ T cells	Immunosuppressive; CD8 T cell-derived CD73^+^ EVs suppress T cell proliferation and cytokine production *in vitro*	(31)
		Activated murine Tregs	Immunosuppressive; adenosine production by Treg-derived CD73^+^ EVs contributes to Treg-mediated suppressive activity *in vitro*	(26)
		Low-dose IL-2 treatment induced murine Tregs	Immunosuppressive; FoxP3^+^ CD73^+^ EVs delay chronic cardiac allograft rejection *in vivo*	(32)
	Mesenchymal stem cells	Human umbilical cord MSC	Immunosuppressive; adenosine production by MSC-derived EVs in cooperation with activated T cells *in vitro*	(33)
		Human bone marrow MSC	Induction of immune tolerance *in vivo* in a xenogeneic GvHD model by adenosine production	(34)
		Cytokine-stimulated human gingival MSC	Immunosuppressive; EVs enhance M2 macrophage polarization *in vitro*, CD73-dependent	(35, 36)
		Human MSC cell line	M2 macrophage polarization *in vitro*, CD73- and adenosine-dependent	(37)
		Human bone marrow MSCs	CD73 enzymatic activity of MSC EVs does not correlate with their immunomodulatory potential *in vitro*	(38)
CD39	Immune cells	Activated human peripheral Tregs	Immunosuppressive; inhibition of effector T cell proliferation, modulatory effect on cytokine profile, protective *in vivo* in GvHD	(39)
	Cancer cells	Human NSCLC cell lines and primary tumor cells	Immunosuppressive; ATP degradation in CD4 T cells, affecting T cell differentiation *in vitro*	(40)
CD39, CD73	Cancer cells	Human bladder-, colorectal-, prostate- and breast cancer cell line, mesothelioma cell line, pleural fluid from patients with malignant pleural mesothelioma	Immunosuppressive; T cell suppression by tumor-derived CD39^+^ CD73^+^ EVs *in vitro* through adenosine production	(25)
		Plasma of AML patients	Immunosuppressive; adenosine production, inhibition of NK-92 cell proliferation, cytotoxicity, migration and modulation of cytokine expression	(41)
		Human plasma of patients with HNSCC	Adenosine production	(42)
		Glioblastoma cell lines	Immunosuppressive *in vitro*	(43)
		Rat glioma cell line	Anti-proliferative effect on tumor cells, leading to reduction of tumor size *in vivo*	(44)
	Immune cells	Human and mouse B cells, human serum of colon cancer patients	Immunosuppressive; impairment of anti-tumor CD8 T cell responses by adenosine production *in vivo*	(45)
	Stem cells	Human amniotic fluids of 16-17 week pregnant women	Immunosuppressive; intrinsic ATP production and degradation to adenosine, suppression of inflammasome activation in THP-1 cells, A2A receptor-dependent	(46)
CD39, CD73, Adenosine		Human plasma of HNSCC patients, HPV(+) HNSCC cell line	Adenosine production, M2 macrophage polarization *in vitro*, A2B receptor-dependent	(47)
CD39, CD73, CD26, ADA		Human plasma of HNSCC patients	Adenosine production, active purinergic enzymes, increased expression in patients with late stage-HNSCC	(48)
CD39 CD73, CD38, ENPP1		Human bone marrow plasma of patients with multiple myeloma	Adenosine production, increased expression in patients with multiple myeloma than in controls	(49)
		Human bone marrow plasma of patients with neuroblastoma	Immunosuppressive; adenosine production and suppression of T cell proliferation *in vitro*, increased expression in patients with worse prognosis	(50)
CD39 and other NTPDases		Human plasma	ADPase activity	(51)
P2X7	Immune cells	Human Bz-ATP-stimulated DCs	P2X7-dependent lysis of the vesicles and release of pro-inflammatory cytokines	(52)
Adenosine	Tumor cells	Human breast cancer cell line	Immunosuppressive; inhibition of cytotoxic T cell effector function *in vitro*	(21)
Adenosine, AMP	Tumor cells	Human HNSCC cell lines, plasma of patients with HNSCC	Changes in EV-contained purine metabolites correlate with tumor progression	(22)
ATP	Tumor microenvironment	Murine melanoma and colon carcinoma tumors	Release of ATP-loaded microparticles and naked mitochondria upon nutrient deprivation	(23)

AML, acute myeloid leukemia; DC, Dendritic cell, GvHD, graft vs. host disease; HNSCC, head and neck squamous cell carcinoma; M2, M2 macrophage; MSC, mesenchymal stem cell; NSCLC, non-small-cell lung cancer.

Beyond CD39 and CD73, other ectonucleotidases such as CD38, ectonucleotide pyrophosphatase/phosphodiesterase 1 (ENPP1/CD203a), alkaline phosphatases (AP), and CD26, as well as purinergic P2 receptors are present on EVs (**Table 1**). CD38 degrades nicotinamide adenine dinucleotide (NAD^+^) to the second messengers ADP-ribose, cyclic ADP-ribose, and NAADP, which modulate the immune response by intracellular Ca^2+^ mobilization from the endoplasmic reticulum (55, 56). CD38 was found on EVs derived from the plasma or bone marrow of patients with multiple myeloma and neuroblastoma, and on EVs from lymphoblastoid B cells (49, 50, 57, 58). ADP-ribose, generated by CD38, can be further metabolized to AMP by ENPP1 (59). In addition, ENPP1 degrades ATP and the STING agonist 2’3’-cGAMP, which is why ENPP1 is considered a modulator of the type I interferon pathway (60). ENPP1-containing EVs were isolated from the bone marrow of patients with multiple myeloma (49). Alkaline phosphatases (APs) are a class of enzymes with broad substrate specificity that dephosphorylate a variety of targets including ATP, ADP and AMP. AP-containing EVs have been found for example in the blood and placenta of pregnant women (61, 62). CD26 is a membrane-bound protein with own enzymatic activity (dipeptidylpeptidase) that serves as an anchor for ADA, the enzyme degrading adenosine to inosine. Its presence on EVs, as shown on tubular epithelial cell-derived EVs after ischemia-reperfusion injury (63), could ‘catch’ soluble ADA and bring it in proximity to target cells. A potential immune modulatory effect of EVs containing these ‘non-canonical’ ectonucleotidases has only rarely been investigated. Notably, stimulation of the P2X7 receptor with ATP induces the release of EVs from dendritic cells (DCs), microglia and tumor cells (52, 64–66). ATP-induced EVs from DCs contain IL-1β as well as the P2X7 receptor itself. Subsequent exposure of the vesicles to ATP results in a P2X7-dependent lysis of the vesicles and the release of cytokines (52).

There are reports of ´soluble´ ENPP1 (67), CD26 (68), CD73, AP, ADA (69, 70), or corresponding enzymatic activities in different body fluids without having specifically differentiated between EVs and the soluble forms of these proteins. Because the EV purification methodology has only recently been standardized, the immune regulatory potential of EVs carrying purinergic enzymes and metabolites is likely higher than the current state of research might suggest. Future studies need to clarify the origin of ´soluble´ purinergic molecules and their role in inflammation.

## 3 Extracellular vesicles as modifiers of cell functions

EVs may be taken up by the target cell by endocytosis or phagocytosis and their content released intracellularly. The molecules delivered by the EVs can modulate cellular functions. Until now, it is not clear to which extent recipient cells take up the cargo of EVs, and how relevant this is for the function of the recipient cell. For many years, the transfer of miRNA and subsequent gene suppression through EVs were seen as a main feature of EV-cell interaction, however, recent publications show that EV uptake is rather a low yield process (71), and that also the transfer of miRNA is low (72). In contrast, vesicular telomere transfer in the immunological synapse occurs very efficiently upon interaction of T cells and antigen-presenting cells, leading to the elongation of telomeres in the T cells and promoting long-term immunological memory (7). It is not yet clear whether the transfer of EV cargo is targeted. In the case of miRNAs, distinct nucleotide motifs determine either secretion in EVs or cellular retention. This indicates an ‘active’ decision of miRNA sorting into EVs (73). By analogy, ATP or adenosine contained in EVs may be released into the cytoplasm of the target cell. As the intracellular concentration of these nucleotides is much higher than in the extracellular space, EV-derived nucleotides will unlikely alter the concentration significantly. Alternatively, adenine nucleotides can be released from EVs in proximity to cells, i.e. adenosine released by cancer EVs reduces cytotoxic T cell perforin secretion, potentially contributing to tumor growth (21).

For EV-bound ectonucleotidases and other membrane proteins that have their active site facing the extracellular space, fusion of the EVs with the target cells is not necessary to exert their function. Proteins in the EV membrane can interact with the target cell, modulating cellular functions. For example, PD-L1 on tumor-derived EVs decreases the proliferation of PD-1-expressing tumor-infiltrating CD8 T cells (74). Additionally, EVs can present antigens, which leads to the activation of immune cells (13, 75). Ectonucleotidases such as CD39, CD73, ENPP1, and APs on EVs metabolize adenine nucleotides in proximity to a target cell without requiring a direct EV-cell contact, i.e. enabling the activation of adenosine receptor signaling on T cells. Considering the extremely short half-life of adenosine of only five to ten seconds (76), the enzymatic activity of EV-bound ectonucleotidases might be essential for immune regulation as it facilitates the production of anti-inflammatory adenosine in proximity to immune cells lacking e.g. CD73 expression. In contrast to soluble enzymes, EV-bound ectonucleotidases could be “attracted” to target cells by EV-cell-interactions and may be more stable, which allows a longer time in circulation. Research to determine the stability and longevity of EVs *in vivo* is still ongoing: When injecting EVs into mice, EVs still could be found after nine days in the lung, liver, and spleen (77), while in another study the EVs were eliminated after six hours (78). In a mouse model with a continuous generation of labeled tumor-EVs *in vivo*, EVs were found 35 days after tumor implantation in almost all organs (79), and breast milk-derived EVs survive circulation through the gastrointestinal tract and reached target organs with miRNA cargo (80). These data show that EVs can travel in the body and that their life span is long enough to reach different organs in the mouse.

## 4 Control of the immune response by extracellular vesicles with purinergic activity

Adenosine binding to the A2A receptor on immune cells modulates antigen presentation, inhibits the activation of T and B cells, suppresses cytokine production, and promotes the generation of Tregs. Such immune regulatory mechanisms help to prevent excessive or prolonged immune responses, which, if not controlled, could lead to tissue damage and autoimmunity. This *a priori* beneficial immune modulatory function has the downside of a potentially premature termination of an immune response against persistent viral antigens and tumors (81).

Adenosine has an extremely short half-life of under 10 seconds. Therefore, the availability of extracellular adenosine depends on the activity of the purinergic enzymes that metabolize ATP to AMP and adenosine. In T cells, both CD39 and CD73 are upregulated shortly after activation, however, while CD39 remains on the cell membrane of activated cells, CD73 is released from the membrane, mostly in EVs (31). In this way, effector cells are able to survive in an ATP-rich environment at the same time that they are not suppressed by pericellular adenosine. EVs containing purinergic enzymes can move away from the cell of origin and exert their enzymatic activity in a paracrine fashion in the vicinity of cells lacking the full machinery for adenosine production. Indeed, human Tregs and Treg-derived EVs are competent in degrading ATP to AMP, but much less efficient in generating adenosine. CD73 can be provided by other cells, either cell-bound or in form of EVs (31, 39). The source of CD73-decorated EVs includes for example recently-activated T cells and stromal cells at sites of inflammation (31, 33), and tumor cells (25) (**Table 1**). In contrast to human Tregs, EVs derived from murine Tregs contain both CD39 and CD73, and are able to generate adenosine by themselves (26).

Hypoxia in the tumor microenvironment triggers the upregulation of CD39 and CD73 in cancer cells (82), which metabolize ATP, generating sufficient adenosine to cause immune suppression (83). Hypoxia also promotes the release of EVs from cancer cells, and these EVs exhibit potent enzymatic activity to degrade ATP and AMP (25) (**Table 1**), suppressing T cell function. B cell-derived EVs carrying CD39 and CD73 further exacerbate the immune suppressive environment within the tumor (45). Both ATP and adenosine have been found in tumor cell-derived EVs (23), and perforin secreted by activated cytotoxic T cells can disrupt the EV membrane and lead to adenosine release that will in turn inhibit cytotoxic responses (21). Antibody-mediated blockade of CD73 enzymatic activity revokes immune suppression by tumor-derived EVs *in vitro*, highlighting the importance of the purinergic pathway in immune suppression in the context of tumors (29). EVs with purinergic enzyme activity travel far beyond the tumor and are detected in peripheral blood, where their presence correlates with cancer progression (30, 45, 49).

In summary, EVs containing purinergic enzyme activity or nucleotides are potent immune modulators whose activity is not restricted to the vicinity of their cells of origin.

## 5 Translational application of extracellular vesicles targeting the purinergic pathway

EVs are promising biomarkers for diagnostics, monitoring of disease course and response to therapy, and already regarded as therapeutic agents. Changes in EV concentration or EV cargo are used as predictive biomarkers for autoimmunity, cancer, ischemia and neurodegeneration. In the field of cancer, the increasing interest in liquid biopsies for the detection of circulating tumor cells provides an excellent platform to test the feasibility of using EVs as predictive biomarkers. Given the high expression of CD73 in tumor cells and its role in immune suppression, especially the presence of CD73 on EVs could help to predict response to immunotherapy (27).

In inflammation, CD73-containing EVs have a beneficial effect by suppressing effector mechanisms or by promoting M2 macrophage polarization and tissue regeneration (35, 47). The advantages of EVs from human cells or cell lines as therapeutic agents are the potential to cross biological barriers, cell-specific function, and their low immunogenicity because they do not display exogenous factors (84, 85). The *in vitro* generation and modulation of mesenchymal stem cell-derived EVs for Covid-19 treatment and autoimmune diseases is currently tested in clinical and pre-clinical studies (86, 87), with no severe adverse effects described so far. In preclinical models, MSC- or Treg-derived EVs have been successfully used in an animal model of rheumatoid arthritis (88, 89). Even though the role of CD73 or adenosine in the modulatory function was not addressed, it is noteworthy that both EV-donor cell types express CD73, and a synergism to the proposed mechanism is plausible.

In order to use EVs for therapy, it is critical to further understand EV biology. Research on EVs has long been hampered due to technical challenges and the lack of standardization protocols for EV isolation, storage and characterization methods. The International Society for Extracellular Vesicles (ISEV) has proposed and updated the “Minimal Information for Studies of Extracellular Vesicles” (MISEV), to promote reproducibility in this field of research (54, 90). EVs can be isolated with increasing purity and recovery yield from various kinds of biological fluids, conditioned cell culture media and tissues (91), facilitating proteomics and transcriptomic analyses (92, 93), as well as enzymatic activity and functional assays (31). Analyses at single-vesicle level are limited due to the small size and high level of heterogeneity of EVs. Nanoscale flow cytometry is promising, but still challenging for surface phenotyping of EVs, as the small size limits the detection and antibody binding (94). While characterization methods are improving, *in vivo* EV tracking remains difficult. Deciphering the physiological stability, biodistribution and interactions of EVs with cells in *in vivo* settings is essential for assessing the (patho)physiological role of EVs and their potential use for therapy. Recent progress in real-time non-invasive imaging, high-resolution microscopy and labelling techniques should pave the way for better understanding of the EV biology.

In conclusion, an increasing number of publications describe the presence of several purinergic enzymes (CD39, CD73, ENPP1, CD26, ADA and APs) as well as of adenine nucleotides (ATP and adenosine) in EVs. Especially on cancer-derived EVs, but also on EVs from immune cells, the adenine nucleotide-metabolizing function of ectonucleotidases has immunomodulatory effects. With advancing technologies, future EV studies will give further insights into the biological role and clinical application of EV-mediated purinergic activity.

## Author contributions

RW: Conceptualization, Writing – original draft, Writing – review & editing. DN: Writing – original draft, Writing – review & editing. FS: Writing – review & editing. FC: Writing – review & editing. NG: Writing – review & editing, Funding acquisition. ET: Writing – review & editing, Conceptualization, Funding acquisition, Supervision, Writing – original draft.
